# Season of Birth and Cardiovascular Mortality in Atrial Fibrillation: A Population-Based Cohort Study

**DOI:** 10.3390/jcdd8120177

**Published:** 2021-12-10

**Authors:** Ying X. Gue, Arnaud Bisson, Alexandre Bodin, Julien Herbert, Gregory Y. H. Lip, Laurent Fauchier

**Affiliations:** 1 Liverpool Centre for Cardiovascular Science, University of Liverpool and Liverpool Heart & Chest Hospital, Liverpool L14 3PE, UK; y.gue@nhs.net (Y.X.G.); gregory.lip@liverpool.ac.uk (G.Y.H.L.); 2 Service de Cardiologie, Centre Hospitalier Universitaire et Faculté de Médecine, Université de Tours, 37044 Tours, France; arnaud.bisson37@gmail.com (A.B.); alexandrebodin.mail@gmail.com (A.B.); j.herbert@chu-tours.fr (J.H.); 3 Service d’Information Médicale, d’Épidémiologie et d’Économie de la Santé, Centre Hospitalier Universitaire et Faculté de Médecine, EA7505, Université de Tours, 37044 Tours, France

**Keywords:** season of birth, atrial fibrillation, mortality, stroke

## Abstract

Background: The fetal origins hypothesis have associated early life exposures with the development of adverse health outcomes in adulthood. Season of birth has been shown to be associated with overall and cardiovascular mortality. Methods: We performed a retrospective database study to explore the association between season of birth and mortality in patients with atrial fibrillation. Results: A total of 8962 patients with AF were identified in the database with 1253 deaths recorded. AF patients born in spring and summer had a higher mortality rate when compared to those born in autumn and winter (hazard ratio (HR) 1.13, 95% confidence interval (CI) 1.01–1.26, *p* = 0.03). This effect was consistent in the male subgroup (HR 1.25, 95% CI 1.03–1.51, *p* = 0.02 for males born in spring; HR 1.24, 95% CI 1.03–1.51, *p* = 0.03 for males born in summer when compared to winter as the reference) but not in females (HR 1.02, 95% CI 0.79–1.31, *p* = 0.88 for females born in spring; HR 1.11, 95% CI 0.87–1.42, *p* = 0.39 for females born in summer when compared to winter as the reference). Results persisted after adjustment for baseline characteristics and clinical risk profile. A similar pattern was observed with cardiovascular mortality. Conclusion: Birth in spring or summer is associated with a higher risk of cardiovascular mortality in male AF patients, but not in females. This could be related to the underlying differences in rates of major adverse clinical events between genders. Further studies should aim at clarifying the mechanisms behind this association, which may help us understand the higher level of risk in female patients with AF.

## 1. Introduction

The fetal origins hypothesis [1,2] have associated early life exposures with the development of adverse health outcomes in adulthood. Season of birth represent proxies for various complex environmental, familial and socioeconomic factors in prenatal and early postnatal life which could explain the association with disease development in adulthood. Season of birth has been shown in multiple studies from different parts of the world to be significantly associated with overall and cardiovascular disease-related mortality [3,4,5,6,7].

It has been suggested that people born in the autumn in the northern hemisphere live longer than those born during the spring or summer, as they have a decreased risk of cardiovascular disease specific mortality [6]. Not only is there an association with cardiovascular mortality, there has been associations of birth seasons on hypertension [8], one of the main contributory disease to cardiovascular mortality and morbidity worldwide. Most of these reported studies are whole population-based and only a few studies followed populations longitudinally [9]. Moreover, none of the studies investigated the relationship between season and month of birth and mortality in patients with established cardiac conditions.

Atrial fibrillation (AF) is the most common sustained cardiac arrhythmia and is associated with an increased risk of major adverse cardiovascular events (MACE) [10]. The complex pathophysiology of atrial fibrillation (AF) encompasses many different mechanisms [11,12] and has been associated with modifiable risk factors such as hypertension and non-modifiable risk factors such as genetics and age [13]. Low birth weight has also previously been associated as a risk factor for development of AF, independent of known predictors such as hypertension, body mass index and height [14]. However, it is unknown whether the season of birth is associated with mortality in patients who develop AF.

This complex interaction between environmental and genetic factors, paired with the strong association of season and month of birth with cardiovascular mortality, makes AF an interesting disease to investigate in order to decipher the potential relationship in mortality between AF and the month and season of birth. This study aims to identify the relationship between mortality and season of birth in patients with AF.

## 2. Methods

We performed a retrospective database analysis of consecutive patients with AF seen in the cardiology department in our institution between January 2000 and December 2010 were identified through a database [15,16,17,18]. Our hospital covers an area of 4000 km^2^, and a population of 400,000 inhabitants and is the only public institution in the area. 

Following identification of eligible patients, basic demographics (age at consultation, gender and season of birth), medical and drug history, biomarkers (left ventricular function, brain natriuretic peptic (BNP) and estimated glomerular filtration rate [19]) and clinical scores including CHA2DS2VASc, HASBLED, Charlson comorbidity index [20], frailty index [21], New York Heart Association (NYHA) and European Heart Rhythm Association (EHRA) functional class were recorded. 

During follow-up, deaths from all causes and events of interest were recorded whenever they occurred in our institution. In addition, mortality data were obtained using a search tool from a dedicated website for the Région Centre (http://nrco.lanouvellerepublique.fr/dossiers/necro/index.php). The causes of death occurring in-hospital were collected through computerized hospitalization reports and were investigated to separate between cardiovascular and non-cardiovascular causes. For those that occurred outside our institution, they were collected by telephone from treating physicians, retirement homes or families. Mode of death was adjudicated based on medical reports and autopsy reports or death certificates when available. This information was reviewed by 2 investigators and causes of death were adjudicated after consideration of all the available information, and according to the following prespecified groups: cardiovascular, non-cardiovascular, as well as unknown when the quality of the information could not allow the investigators to appropriately identify cause of death [15]. The relationship between season of birth (autumn, winter, spring and summer) and mortality risk (all-cause and cardiovascular) was assessed using Cox proportional hazard regression models using autumn as the reference and separate analyses were performed for men and women as a gender subgroup. 

Further regression analysis was performed using models to adjust for baseline characteristics and clinical risk profile.

The study was approved by the institutional review board of the Pole Coeur Thorax Vaisseaux from the Trousseau University Hospital, on 1 December 2015 and registered as a clinical audit. Ethical review was therefore not required. Patient consent was not sought. The study was conducted retrospectively, patients were not involved in its conduct, and there was no impact on their care.

## 3. Results

A total of 8962 patients with AF were identified in the database. The mean age was 70.8 ± 14.6 years with 61.3% males with a follow-up period of 2.5 ± 3.0 years (median 1.2, interquartile 4.3 years, yearly rate of death 5.5%). Demographics, medical history and clinical scores were similar between the different seasons, as shown in Table 1. 

Within this cohort, a total of 1253 deaths were recorded (1114 in-hospital deaths and 139 out-of-hospital deaths) during the follow-up period and mode of death was identified in 1215 (97%) patients (1080 (97%) for in-hospital deaths and 135 (97%) for out-of-hospital deaths). Of these, (677) 54% was attributed to cardiovascular causes whereas 538 (43%) were non-cardiovascular related causes. The three main causes of death were heart failure (29%), infection (18%) and cancer (12%). There were 715 strokes or thromboembolic events (TE) recorded during follow-up.

### 3.1. Gender Subgroup

When comparing between the 2 genders, the distribution of season of birth was similar as shown in Table 2. There were significant differences between the baseline characteristics of the 2 genders, with females being older and have a lower proportion of with concomitant cardiovascular risk factors and comorbidities (Table 2).

### 3.2. All-Cause Mortality

**Figure 1:**
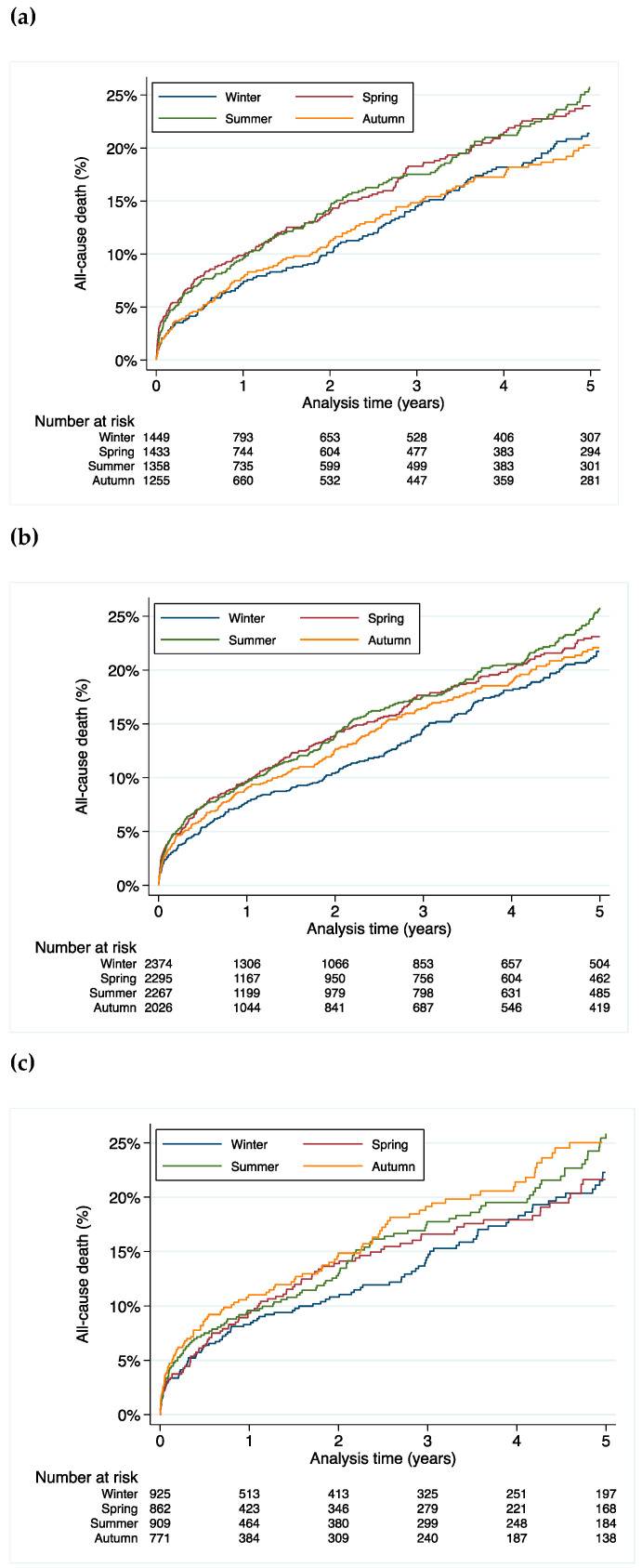
All-cause mortality stratified by season of birth for (**a**) all patients, (**b**) male patients and (**c**) female patients.

People born in spring and summer had a higher mortality rate when compared to those born in autumn and winter (hazard ratio (HR) 1.13, 95% confidence interval (CI) 1.01–1.26, *p* = 0.03) (Figure 1a).

In the subgroup analysis, the difference in mortality was driven by a higher mortality in males (HR 1.25, 95% CI 1.03–1.51, *p* = 0.02 for males born in spring; HR 1.24, 95% CI 1.03–1.51, *p* = 0.03 for males born in summer, HR 1.02, 95% CI 0.83–1.26, *p* = 0.84 for males born in autumn when compared to winter as the reference) (Figure 1b). This effect was not statistically significant in females (HR 1.02, 95% CI 0.79–1.31, *p* = 0.88 for females born in spring; HR 1.11, 95% CI 0.87–1.42, *p* = 0.39 for females born in summer, HR 1.22, 95% CI 0.95–1.57, *p* = 0.12 for females born in autumn when compared to winter as the reference) (Figure 1c).

### 3.3. Cardiovascular Mortality

**Figure 2:**
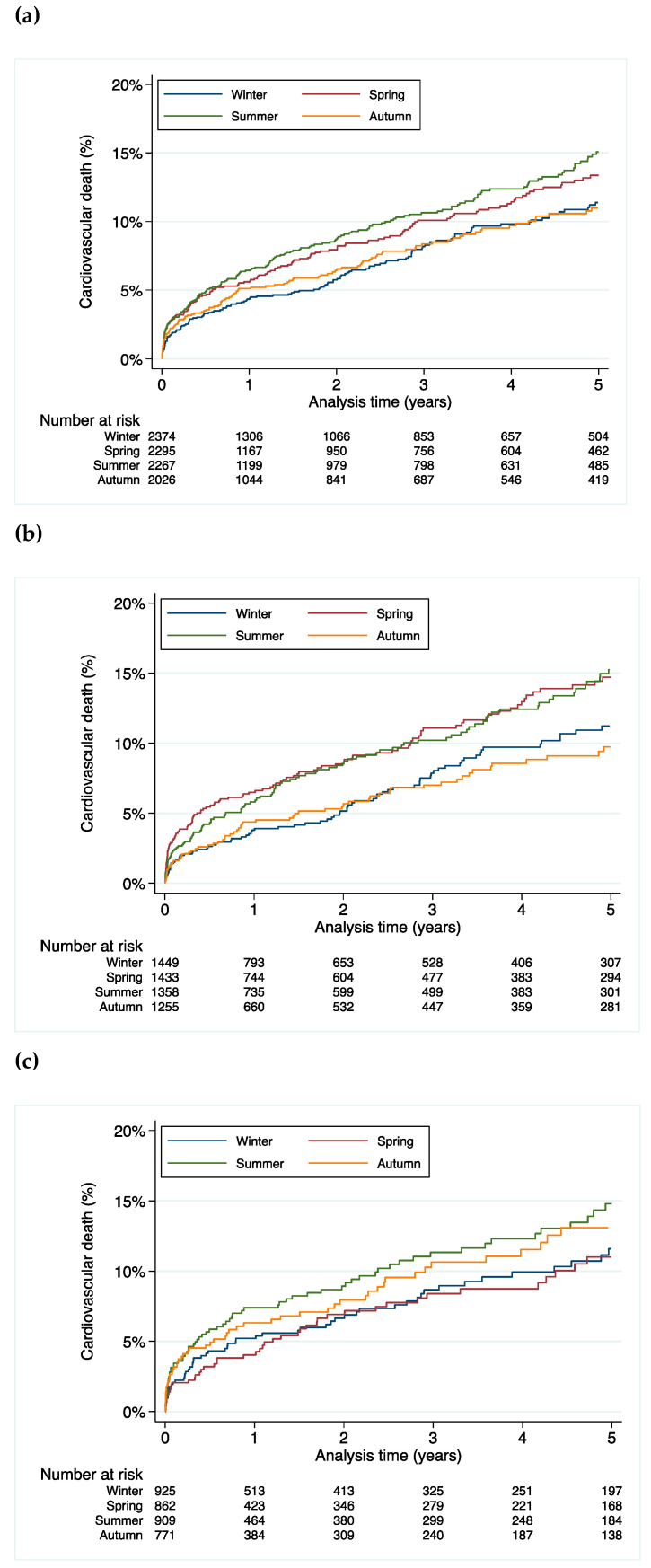
Cardiovascular mortality stratified by season of birth for (**a**) all patients, (**b**) male patients and (**c**) female patients.

Season of birth was a significant predictor for cardiovascular mortality with mortality rate being highest in the cohort born in spring and summer (HR 1.26, 95% CI 1.08–1.45, *p* = 0.003 compared to the born in autumn and winter) (Figure 2a).

Similarly, the difference in mortality was driven by the higher cardiovascular mortality in males (HR 1.41, 95% CI 1.08–1.83, *p* = 0.01 for males born in spring; HR 1.39, 95% CI 1.07–1.81, *p* = 0.02 for males born in summer, HR 0.96, 95% CI 0.72–1.30, *p* = 0.80 for males born in autumn when compared to winter as the reference) (Figure 2b). This effect was not statistically significant in females (HR 0.92, 95% CI 0.65–1.31, *p* = 0.63 for females born in spring; HR 1.21, 95% CI 0.88–1.67, *p* = 0.24 for females born in summer, HR 1.11, 95% CI 0.79–1.56, *p* = 0.56 for females born in autumn when compared to winter as the reference) (Figure 2c).

### 3.4. Ischemic Stroke and Thromboembolic Events

Season of birth was not a significant predictor for stroke/TE in AF patients born in spring and summer compared to those born in autumn and winter (HR 0.99, 95% CI 0.86–1.15, *p* = 0.91). 

### 3.5. Multivariable Analysis 

After adjustment for age, CHA2DS2VASc score, HASBLED score, cardiovascular risk factors, other comorbidities, AF pattern, antithrombotic use and other cardiovascular drugs use, a higher all-cause mortality was still seen in males born in spring or in summer (adjusted HR 1.19, 95% CI 1.02–1.38, *p* = 0.03 vs those born in autumn or in winter) while this was not seen in females (adjusted HR 1.02, 95% CI 0.83–1.24, *p* = 0.88 for females born in spring or in summer vs those born in autumn or in winter)

Similarly, a higher cardiovascular mortality was seen in males born in spring or in summer (adjusted HR 1.38, 95% CI 1.12–1.70, *p* = 0.002 vs those born in autumn or in winter) while this was not seen in females (adjusted HR 1.02, 95% CI 0.78–1.34, *p* = 0.89 for females born in spring or in summer vs those born in autumn or in winter).

There was no significant difference when considering the risk of stroke/TE (adjusted HR 1.11, 95% CI 0.90–1.36, *p* = 0.34 for males born in spring or in summer vs those born in autumn or in winter; adjusted HR 0.96, 95% CI 0.75–1.22, *p* = 0.74 for females born in spring or in summer vs those born in autumn or in winter).

There was no statistical interaction when taking into account the early (2000–2004) or later phase (2005–2010) of the analysis: *p* value for interaction 0.89 in subgroups 2000–2004 (*n* = 4237) vs 2005–2010 (*n* = 4725) for the risk of all-cause death when comparing AF patients born in spring or in summer vs those born in autumn or in winter, *p* value for interaction 0.30 for the risk of cardiovascular death and *p* value for interaction 0.90 for the risk of stroke/TE.

## 4. Discussion

This study has shown an association between birth season and cardiovascular mortality in patients with AF, which is consistent with prior evidence in large population-based studies in undifferentiated cohort of patients [5]. This lends some support to the fetal origin hypothesis in the AF population. Where our AF cohort differed from an undifferentiated population was that the impact of season of birth on both all-cause and cardiovascular mortality was absent in the female subgroup. Even after adjusting for potential confounding factors, season of birth remains an independent risk factor for mortality only in males with AF. Our results could relate to a small but real seasonal effect of foetal or early life factors in later life, which should be better investigated and possibly addressed in case modifiable risk factors are identified during this specific period regarding the subsequent risk of cardiovascular events.

Our findings conflict with a previous prospective, longitudinal study, which looked at cardiovascular disease mortality in a non-selective cohort of women in the US and has shown an increase in cardiovascular mortality after adjustment of confounders such as age, familial and socioeconomic factors [9]. This may be due to the much smaller sample size and hence it is underpowered to determine the actual effect. However, it could also signify that the presence of AF in females negates the impact that birth season has on cardiovascular mortality. 

Sex differences in AF have been previously been known and recognised [22], in particular the higher risk of stroke in females when compared to males [23]. This difference in stroke risk remains present despite treatment with oral anticoagulation (vitamin K antagonists) although it is nullified with the use of non-vitamin K antagonist oral anticoagulants [24]. A potential explanation could be differences in the clinical risk profile when female patients develop AF, being at a slightly higher risk of stroke and systemic embolism than their male counterparts, which negates the potentially beneficial or protective effect of being born in the autumn or winter months that can be seen in males. Although low birth weight has been implicated as a potential risk factor for stroke [25], it has not been explored as an outcome when comparing season of birth. As only half the population was on oral anticoagulation and there were no data on the time in therapeutic range, the interpretation of the results on stroke/TE is limited. The absence of significant differences in stroke/TE, a major driver of mortality in non-anticoagulated AF population, renders difficulties in the interpretation of the mortality results. Further studies are needed to examine the mechanisms behind this association.

Season of birth has been shown in previous studies to be significantly associated with cardiovascular disease-related mortality [3,4,5,6,7]. Potential drivers for this observed phenomenon includes dietary and nutritional differences, which could be related to the availability of types of food at different seasons and socioeconomic factors [2,26], inflammation and infective causes [27], environmental and climate differences [28]. The findings from this study may have important clinical implications for the future. Firstly, populations at a higher risk of cardiovascular disease may benefit from closer monitoring to detect early development of cardiovascular disease and initiate early, aggressive primary prevention measures to reduce mortality. Secondly, it may stimulate research into identifying the reasons behind the higher risk. As previously mentioned, the combination of complex environmental, familial and socioeconomic factors likely contributes to this association and to being able to identify and isolate certain modifiable factors which may allow cardiovascular risk prevention to start, even before birth. 

### Limitations

One of the limitations is the small sample size when compared to other population-based studies exploring birth season and mortality. This may impact on the effect size and may be underpowered to identify small differences, such as in the female subgroup. Secondly, being a single centre study reduces the generalizability of the results, although the overall conclusion appears to correlate with other published data. Similarly, as the population in the analysis is comprised mainly of pre non-vitamin K antagonists oral anticoagulant (NOAC), the results may not be present in contemporary cohort of patients on NOAC. In the same note, considering the mean age of the patients, the conditions surrounding their birth years and will very unlikely be replicated in the future and hence may not be extrapolated. Third, the lack of data from birth such as birth weight, which could be a confounder, may limit the conclusions drawn. Fourth, the date of diagnosis of AF is unknown which could have an impact on the analysis. Lastly, a major limitation to these findings and that of the fetal origins hypothesis is the inadequate control for some early and later life predictors of mortality which may contribute significantly to variation in lifetime health conditions [1,29]. An example would be that socioeconomic factors could be a confounder as women in more favourable socioeconomic status chose to avoid giving birth in colder months [3,30]. These were not investigated in our study and hence could not be accounted for within the analyses.

## 5. Conclusions

Birth in spring or summer is associated with a higher risk of cardiovascular mortality in male AF patients, consistent with other similar studies of undifferentiated cohort. However, this effect was not seen in females which could be related to the underlying differences in rates of major adverse clinical events between genders.

## 6. Learning Points

1. Season of birth is associated with both overall and cardiovascular mortality.

2. Birth in spring or summer is associated with a higher risk of cardiovascular mortality in male AF patients, but not in females.

3. This could be related to the underlying differences in the rates of major adverse clinical events between genders but more studies are required to confirm this.

## Figures and Tables

**Table 1:** Baseline characteristics according to season of birth.

	Winter	Spring	Summer	Autumn	All Patients
	(*n* = 2374)	(*n* = 2295)	(*n* = 2267)	(*n* = 2026)	(*n* = 8962)
Age, years	70.9 ± 14.7	70.8 ± 14.1	70.9 ± 14.9	70.4 ± 14.7	70.8 ± 14.6
Sex (male)	1449 (61.0)	1433 (62.4)	1358 (59.9)	1255 (61.9)	5495 (61.3)
Hypertension	901 (38.0)	884 (38.5)	831 (36.7)	748 (36.9)	3364 (37.5)
Diabetes mellitus	329 (13.9)	338 (14.7)	292 (12.9)	273 (13.5)	1232 (13.8)
Dyslipidemia	398 (16.8)	381 (16.6)	388 (17.1)	337 (16.6)	1504 (16.8)
Smoker	262 (11.0)	265 (11.5)	278 (12.3)	206 (10.2)	1011 (11.3)
Obesity	358 (15.1)	340 (14.8)	310 (13.7)	269 (13.3)	1277 (14.3)
Alcohol related diagnoses	66 (2.8)	69 (3.0)	81 (3.6)	45 (2.2)	261 (2.9)
Heart failure	1197 (50.4)	1106 (48.2)	1130 (49.8)	990 (48.9)	4423 (49.4)
Coronary artery disease	636 (26.8)	644 (28.1)	663 (29.2)	564 (27.8)	2507 (28.0)
Previous stroke	182 (7.7)	200 (8.7)	171 (7.5)	158 (7.8)	711 (7.9)
Vascular disease	468 (19.7)	462 (20.1)	457 (20.2)	391 (19.3)	1778 (19.8)
Chronic kidney disease	154 (6.5)	151 (6.6)	153 (6.7)	117 (5.8)	575 (6.4)
Liver disease	7 (0.3)	6 (0.3)	6 (0.3)	2 (0.1)	21 (0.2)
Chronic lung disease	184 (7.8)	230 (10.0)	183 (8.1)	161 (7.9)	758 (8.5)
Permanent AF	930 (39.2)	895 (39.0)	876 (38.6)	794 (39.2)	3495 (39.0)
Left BBB	110 (4.6)	111 (4.8)	101 (4.5)	99 (4.9)	421 (4.7)
Right BBB	112 (4.7)	100 (4.4)	107 (4.7)	80 (3.9)	399 (4.5)
Previous pacemaker or ICD	320 (13.5)	331 (14.4)	360 (15.9)	314 (15.5)	1325 (14.8)
CHA2DS2VASc score	2.9 ± 1.7	2.9 ± 1.7	2.9 ± 1.7	2.9 ± 1.7	2.9 ± 1.7
HASBLED score	1.5 ± 1.0	1.5 ± 1.0	1.5 ± 1.0	1.5 ± 1.0	1.5 ± 1.0
Charlson comorbidity index	1.0 ± 1.3	1.0 ± 1.3	1.0 ± 1.3	1.0 ± 1.3	1.0 ± 1.3
Frailty index	0.7 ± 0.6	0.7 ± 0.6	0.7 ± 0.6	0.7 ± 0.6	0.7 ± 0.6
NYHA functional class	2.0 ± 0.9	2.0 ± 0.9	2.0 ± 0.9	1.9 ± 0.9	2.0 ± 0.9
EHRA functional class	2.1 ± 0.8	2.1 ± 0.8	2.1 ± 0.8	2.0 ± 0.8	2.1 ± 0.8
LVEF, %	47.8 ± 16.0	47.4 ± 15.5	46.7 ± 16.0	46.3 ± 16.2	47.1 ± 15.9
BNP, pg/mL	573.0 ± 838.1	644.3 ± 977.9	617.8 ± 764.3	582.4 ± 665.5	605.4 ± 823.2
eGFR, mL/min/1.73 m^2^	62.4 ± 20.3	62.4 ± 20.4	62.2 ± 20.0	63.1 ± 34.4	62.6 ± 24.2
VKA	1217 (56.6)	1224 (58.6)	1202 (58.5)	995 (54.5)	4638 (57.1)
Antiplatelet therapy	712 (33.8)	688 (33.7)	646 (31.9)	638 (35.6)	2684 (33.7)
ACEi/ARB-II	770 (33.5)	778 (35.0)	753 (34.4)	679 (34.6)	2980 (34.4)
Beta Blocker	1008 (43.3)	934 (41.6)	966 (43.7)	881 (44.4)	3789 (43.2)
Digoxin	526 (22.4)	518 (22.7)	519 (23.1)	484 (24.2)	2047 (23.1)
Diuretic	859 (39.2)	818 (39.4)	839 (40.3)	773 (41.3)	3289 (40.0)
Antiarrhythmic agent	757 (53.7)	700 (53.0)	712 (52.1)	662 (54.8)	2831 (53.4)

Values are *n* (%) or mean ± SD. AF—Atrial Fibrillation; ACEi—Angiotensin converting enzyme inhibitor; ARB-II—angiotensin 2 receptor blockers; BBB—Bundle branch block; BNP—brain natriuretic peptide; eGFR—estimated glomerular filtration rate; ICD—Implantable cardioverter-defibrillator; LVEF—Left ventricular ejection fraction; SD = standard deviation; VKA—Vitamin K antagonists.

**Table 2:** Baseline characteristics of patients with AF according to sex.

	Women	Men	*p*
	(*n* = 3467)	(*n* = 5495)	
Age, years	73.9 ± 14.6	68.8 ± 14.2	< 0.0001
Hypertension	1472 (42.5)	1892 (34.4)	<0.0001
Diabetes mellitus	455 (13.1)	777 (14.1)	0.17
Dyslipidemia	524 (15.1)	980 (17.8)	0.001
Smoker	114 (3.3)	897 (16.3)	<0.0001
Obesity	421 (12.1)	856 (15.6)	<0.0001
Alcohol related diagnoses	31 (0.9)	230 (4.2)	<0.0001
Heart failure	1779 (51.3)	2644 (48.1)	0.003
Coronary artery disease	685 (19.8)	1822 (33.2)	<0.0001
Previous stroke	288 (8.3)	423 (7.7)	0.3
Vascular disease	502 (14.5)	1276 (23.2)	<0.0001
Chronic kidney disease	202 (5.8)	373 (6.8)	0.07
Liver disease	2 (0.1)	19 (0.3)	0.01
Chronic lung disease	234 (6.7)	524 (9.5)	<0.0001
Permanent AF	1183 (34.1)	2312 (42.1)	<0.0001
Left BBB	130 (3.7)	291 (5.3)	0.001
Right BBB	120 (3.5)	279 (5.1)	0.0003
Previous pacemaker or ICD	382 (11.0)	943 (17.2)	<0.0001
CHA2DS2VASc score	3.7 ± 1.6	2.4 ± 1.6	<0.0001
HASBLED score	1.6 ± 1.0	1.4 ± 1.1	<0.0001
Charlson comorbidity index	0.9 ± 1.2	1.1 ± 1.4	<0.0001
Frailty index	0.8 ± 0.6	0.6 ± 0.6	<0.0001
NYHA functional class	2.0 ± 0.9	1.9 ± 0.9	0.02
EHRA functional class	2.1 ± 0.8	2.0 ± 0.8	0.004
LVEF, %	52.6 ± 15.0	43.8 ± 15.5	<0.0001
BNP, pg/mL	593.5 ± 851.1	611.9 ± 806.0	0.58
eGFR, mL/min/1.73 m^2^	56.8 ± 18.8	66.1 ± 26.3	<0.0001
VKA	1630 (52.1)	3008 (60.3)	<0.0001
Antiplatelet therapy	1025 (33.4)	1659 (33.8)	0.71
ACEi/ARB-II	960 (28.7)	2020 (38.0)	<0.0001
Beta Blocker	1481 (43.7)	2308 (42.9)	0.51
Digoxin	900 (26.2)	1147 (21.1)	<0.0001
Diuretic	1337 (40.9)	1952 (39.4)	0.2
Antiarrhythmic agent	1197 (55.3)	1634 (52.0)	0.02
Born in Spring	862 (24.9)	1433 (26.1)	0.2
Born in Summer	909 (26.2)	1358 (24.7)	0.11
Born in Autumn	771 (22.2)	1255 (22.8)	0.51
Born in Winter	925 (26.7)	1449 (26.4)	0.75

Values are *n* (%) or mean ± SD. AF—Atrial Fibrillation; ACEi—Angiotensin converting enzyme inhibitor; ARB-II—angiotensin 2 receptor blockers; BBB—Bundle branch block; BNP—brain natriuretic peptide; eGFR—estimated glomerular filtration rate; ICD—Implantable cardioverter-defibrillator; LVEF—Left ventricular ejection fraction; SD = standard deviation; VKA—Vitamin K antagonists.

## Data Availability

The data presented in this study are available on request from the corresponding author.
